# The Increasing Predictive Validity of Self-Rated Health

**DOI:** 10.1371/journal.pone.0084933

**Published:** 2014-01-22

**Authors:** Jason Schnittker, Valerio Bacak

**Affiliations:** University of Pennsylvania, Department of Sociology, Philadelphia, Pennsylvania, United States of America; University of California, United States of America

## Abstract

Using the 1980 to 2002 General Social Survey, a repeated cross-sectional study that has been linked to the National Death Index through 2008, this study examines the changing relationship between self-rated health and mortality. Research has established that self-rated health has exceptional predictive validity with respect to mortality, but this validity may be deteriorating in light of the rapid medicalization of seemingly superficial conditions and increasingly high expectations for good health. Yet the current study shows the validity of self-rated health is increasing over time. Individuals are apparently better at assessing their health in 2002 than they were in 1980 and, for this reason, the relationship between self-rated health and mortality is considerably stronger across all levels of self-rated health. Several potential mechanisms for this increase are explored. More schooling and more cognitive ability increase the predictive validity of self-rated health, but neither of these influences explains the growing association between self-rated health and mortality. The association is also invariant to changing causes of death, including a decline in accidental deaths, which are, by definition, unanticipated by the individual. Using data from the final two waves of data, we find suggestive evidence that exposure to more health information is the driving force, but we also show that the source of information is very important. For example, the relationship between self-rated health and mortality is smaller among those who use the internet to find health information than among those who do not.

## Introduction

Despite its seemingly superficial character, self-rated health has been shown to be an unusually strong predictor of mortality [1], [2]. A recent meta-analysis finds those who report “poor” health have a twofold higher risk of all-cause mortality relative to those who report “excellent” health [3]. Furthermore, this relationship persists when adjusting for more objective indicators of health, including multiple diagnosed diseases, biomarkers, and functional abilities. This unusually robust relationship is surprising if one believes self-rated health is based on individual perception rather than objective assessment or that individuals systematically misreport their health in ways that dilute its value. Yet the intuition that self-reporting diminishes accuracy is only side of the coin: the strength of self-rated health, in part, reflects its lack of definition. When asked to rate their health individuals consider a more inclusive set of factors than is usually possible to include in a survey instrument or even to gather in a routine clinical examination [4].

In light of vast changes in health and information technology over time, it is now time to expand the focus of the literature. If the validity of self-rated health is premised on accurately evaluating the many relevant dimensions of health, it is likely the relationship between self-rated health and mortality has changed over time. If so, however, it is not entirely clear *how* the relationship has changed and there are perhaps just as many reasons to anticipate a deteriorating relationship as a strengthening one. Some scholars fear, for example, a growing contamination of self-rated health by the widespread medicalization of seemingly superficial conditions [5] or by potential overdiagnosis more generally [6]. Expectations for health have generally increased over time, meaning individuals set a lower bar for reporting “poor” health [7]. Furthermore, it is increasingly difficult to reach a cultural consensus regarding what is or is not disease, potentially allowing assessments of poor health to include a variety of symptoms only weakly related to disease and mortality [8]. In this light, individuals may be objectively healthier than before but feel sicker and deflate their self-rated health accordingly.

In this study, we investigate the changing intersection of self-rated health and mortality. We investigate trends in the relationship between self-rated health and mortality using a repeated cross-sectional survey conducted in the United States from 1980 to 2002 and linked to mortality records through 2008 [9]. The survey provides an unprecedented opportunity. In addition to allowing us to investigate trends, the data allow us consider whether certain sources of health-related information (e.g., the internet versus a physician) have been especially influential. We are also able consider more general influences reflecting a more intellectually sophisticated public, including rising levels of educational attainment and cognitive sophistication, thereby situating self-rated health within the larger context of modernization, medicalization, and culture.

### Background

The validity of self-rated health reflects an accurate understanding of health, but in assessing trends in that validity, there are numerous countervailing influences to consider. One influence is simply increasing exposure to health-related information, both in clinical and public settings. There are more tests available in routine clinical exams, as well as more information regarding risks that are readily apparent to the individual apart from exams, including obesity, diet, exercise, and other health behaviors (see [10] on the “activated” patient). This increase in information is not only a reflection of technology or knowledge. Patients are demanding more information from their physicians than they did in the past [11] and physicians are more inclined to use screening [12], [13]. These trends are likely relevant to the relationship between self-rated health and mortality. For instance, research has shown that individuals who have had contact with the health care system provide more valid reports of self-rated health than those with no such contact [14]. If individuals are getting more information from routine visits, as well as from other sources, the validity of self-rated health may be increasing.

These patterns have a parallel in popular media, but in that context the implications are less clear. In print media, direct-to-consumer advertising of pharmaceuticals has increased steadily since 1989, with an especially sharp increase in television advertising in the late 1990s following changes in broadcast advertising guidelines [15]. Of course, these advertisements primarily serve a promotional function, but they can also serve an educational function in at least two ways, first, by making consumers aware of conditions they might not have recognized before or, second, by making conditions they were aware of more personally salient (see [16] for this and other complexities). In tandem with these trends has been, of course, the emergence of an entirely new source of health information, one perhaps especially well-suited to self-diagnosis. In 2010, 59% of American adults searched for health information online, compared to 25% in 2000 [17]. This trend is perhaps even more powerful than it seems. Health websites are often organized to help the individual reach an appropriate diagnosis, meaning they are structured to help individuals understand symptoms more than they are to educate consumers about what medicine can provide [18].

The public is becoming more sophisticated in other ways as well. Education increases the predictive validity of self-rated health, and average educational attainment in the U.S. has increased over time, especially with respect to high school completion [19]–[22]. Similarly, average scores on measures of cognitive ability have increased, reflecting, according to one interpretation, a public that has responded to the demands of an increasingly complex environment [23], [24]. If the validity of self-rated health rests in part on a discerning reporting of health based on an accurate understanding about what poor health is, its validity may be increasing apace.

Yet the relevance of health information also depends on how individuals interpret the information available to them. In particular, the oft-discussed process of medicalization creates ambiguity surrounding the meaning of “excellent” health. Medicalization is the process by which previously non-medical problems are defined and treated using a medical framework (p. 4) [25]. In the process of medicalization, clusters of symptoms that were once disconnected become newly categorized as disorders and, thereby, interpreted in a medical framework, meaning sensations that might once have been disregarded are newly recognized as symptoms of a coherent illness. The process of medicalization is generally expansive in the sense that it creates new disorders faster than old ones are discarded, but, in recent years, the process has likely accelerated even further [26]. One implication of this is that individuals may regard a wider array of symptoms and conditions as indicative of poor health, regardless of how strongly these conditions are actually related to mortality (see, for example, depression [27] or erectile dysfunction [28]). Furthermore, if there are more treatments available than there were in the past, the public may be less tolerant of even mild declines in functioning. In this vein, direct-to-consumer advertisements are informative in identifying treatments, but they are imperfect instruments for diagnosis. They often fail to specify the prevalence of the condition, meaning they fail to set a baseline, or to disabuse consumers of common misconceptions, meaning they induce more sensitivity if not more specificity [15]. With the right cognitive framework and an abundance of possible symptoms, the potential for reporting poor health is large. In any given month, 75 to 80% of people experience one or more illness or injury, even though most people do not regard these experiences as severe enough to consult a physician [29], [30].

These countervailing influences present a complex picture, but some unusual trends in self-rated health perhaps reflect their influence. For example, while mortality and disability have declined over time [31], self-rated health has changed very little [32] or gotten worse [33]. Building on these discrepancies, Salomon and colleagues [34] express skepticism regarding self-rated health as a measure of population health, but they nonetheless use these discrepancies to illustrate how self-rated health captures important health-related expectations. In the same vein, Sen [35] finds an inverse relationship between self-rated health and longevity among states in India, suggesting a relationship between access to medical facilities and higher self-reported morbidity.

**Figure 1:**
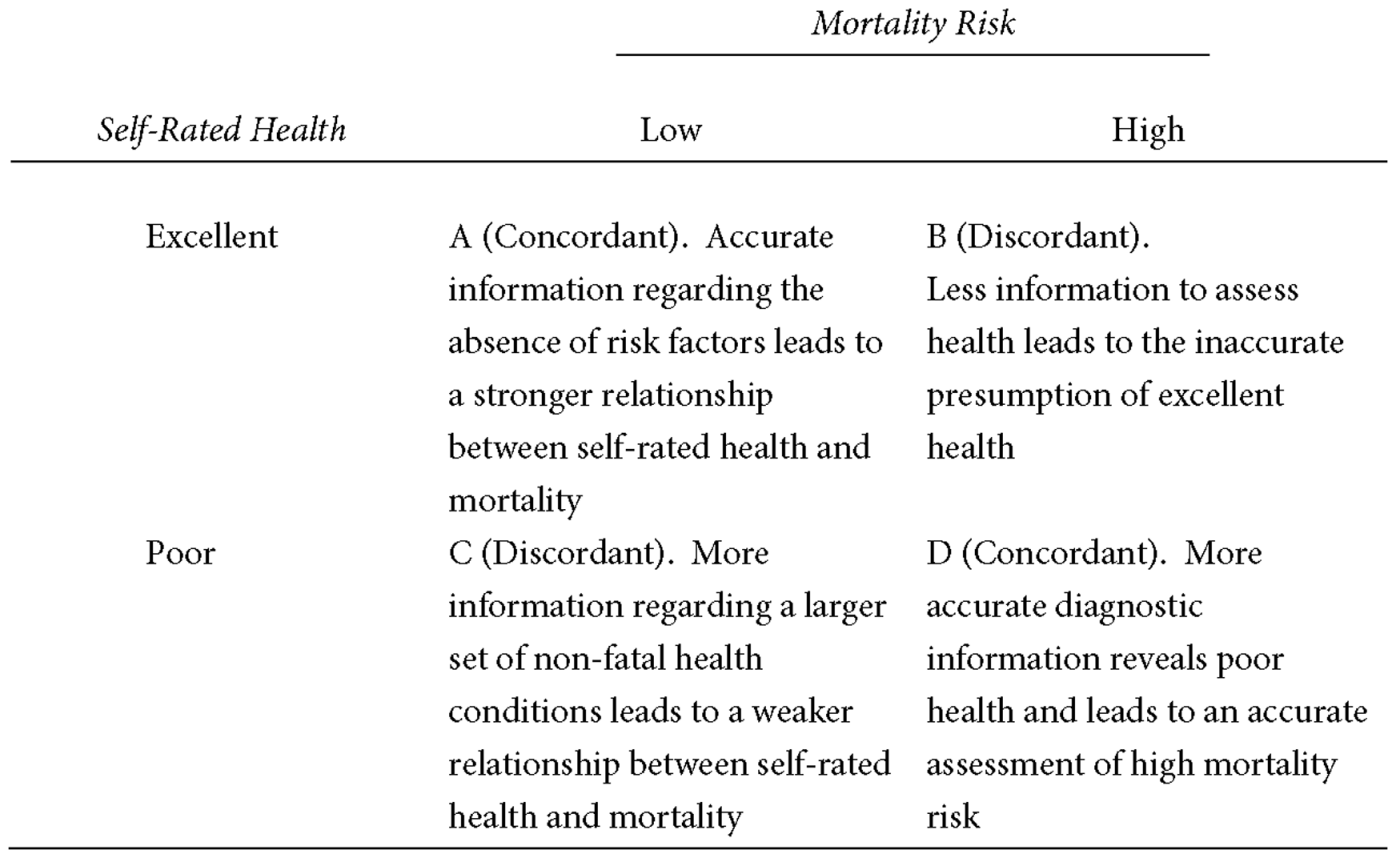
Potential Sources of a Discrepancy (and Concordance) between Self-Rated Health and Mortality Risk.

By way of summary, Figure 1 provides a two-by-two illustration of factors influencing the predictive validity of self-rated health. Quadrants A and D represent “accurate” or “concordant” assessments, reflecting an alignment of self-rated health with objective mortality risk. Quadrants B and C, meanwhile, represent off-diagonal or “discordant” influences, which deteriorate the relationship between self-rated health and mortality by under- or over-stating true risk. Each cell presents examples of relevant influences, across which the indeterminate nature of trends in the predictive validity of self-rated health is illustrated. This situation presents an opportunity for empirical assessment.

## Methods

Data are drawn from a secondary source: the General Social Survey, an ongoing social survey collected by the National Opinion Research Center [36]. The data are available publicly and contain no unique identifiers. The original collectors of the data, the National Opinion Research Center at the University of Chicago, obtained informed consent from all the original participants. The GSS was initiated in the early 1970s and is ongoing. It was originally collected on an annual basis, but in the mid-1990s moved to a biennial schedule. The GSS is primarily a social science and public opinion survey. For this reason it has historically included few health variables apart from self-rated health and some periodic questions on health appearing rotating topical modules. Recently, however, the 1978 to 2002 waves of the GSS were linked to the National Death Index (NDI) through 2008, providing an excellent data source for those interested in mortality [9]. The linkage is ongoing with the intention of eventually linking later waves as well, but at present there are too few deaths in waves subsequent to 2002 to allow for meaningful inferences. In addition to year of death, the NDI linkage provides information on the cause of death, based on the Clinical Classification Software system (which is premised on the ninth and tenth revisions of the International Classification of Disease). The complete NDI linkage is available for 32,830 respondents arrayed over waves—virtually all the GSS respondents during the period—although our analyses use only a subset of respondents for whom the relevant questions were asked. The GSS utilizes a random ballot format, discussed in more detail below, which reduces the analytic sample by approximately half, but does not reduce the representative nature of the data. In the total sample, there were 9,271 deaths. The fraction of deaths across all waves was 28.2%, although, of course, the fraction is much higher among 1978 respondents (45.7%) than 2002 respondents (10.8%).

In the GSS waves used in the linkage, the survey sampled English-speaking non-institutionalized adults using a multi-stage probability framework (a Spanish-language instrument was introduced in 2006). The survey instrument is administered in-person and, in part for this reason, the GSS maintains a consistently high response rate between 70% and 82%. The GSS is nationally-representative by design, although in 1982 and 1987 it included an oversample of African Americans. All the analyses presented here adjust for this oversampling, as well as clustering based on primary sampling units, using a survey weights approach with robust standard errors. We use STATA version 11. The representative nature of the GSS provides an improvement over many of the samples used in previous studies of self-rated health. Some studies have focused on the elderly, for example, limiting their relevance for those interested in the ratings of those in better average health [1], [37].

### Self-Rated Health

Self-rated health is our primary independent variable. Starting in 1980, self-rated health was included for at least a random subset of GSS respondents (it was not asked in 1983 or 1986, but it was asked every other year). In early waves, it was asked among no less than 896 respondents per wave and, in recent waves, among more than 2,000. Respondents were asked, “Would you say your own health, in general, is excellent, good, fair, or poor?” Consistent with previous research, the largest fraction of respondents answered “good,” and relatively few answered “poor.” Although it is common to recode self-rated health as a dichotomy, as “poor” (or fair/poor) versus all other responses, we are concerned with the dose-dependence of self-rated health. We anticipate meaningful shifts between all adjacent categories, but the specificity of the change is not at all clear. For example, a more informed public might meaningfully fluctuate between good and fair health rather than poor and fair health, meaning assessments of trends must be attentive to level-specific changes. For models that use a continuous specification of self-rated health, the variable is coded such that higher values equal worse health.

### Health Behaviors

In the process of establishing an average relationship between self-rated health and mortality, we control for some key health behaviors. This has been done before in other studies, which show that health behaviors explain only part of the self-rated health and mortality relationship, but it is useful to replicate that finding here for purposes of illustration. Unfortunately questions regarding health behavior were only asked during a limited window. Respondents were asked several questions about smoking and drinking, but only from 1980 to 1994. They were asked if they are currently or were ever a smoker, from which we constructed two variables indicating current smoker and previous smoker, both relative to the reference category of never a smoker. Respondents were also asked if they “ever have occasion to use any alcoholic beverages.” Although respondents were also asked if they “sometimes drink more than you think you should,” a variable indicating “excess” drinking did not yield a significant improvement in model fit over a variable indicating only whether the respondent drank or not. These variables are used to demonstrate a robust self-rated health-mortality relationship, although in fewer waves than are used in the remaining models.

### Health Information

The GSS utilizes a supplemental module system in which topical questions not otherwise included in the GSS core are asked among a random subset of respondents in a wave. In 2000 and 2002, a module entitled *Information Society* was administered to approximately half the total sample. The module focuses on the internet in particular, but also includes a series of questions regarding health-related information from a variety of other sources. Respondents were first asked, “In the past year, have you looked for information about a health concern or medical problem?” after which they were asked about seven specific sources: articles in a daily newspaper; articles in a general-interest magazine; special health or medical magazine or newsletter; a doctor, nurse, or other medical professional; friends or relatives; radio or television programs; and the internet or world wide web. For each item, we created a dichotomous variable, indicating whether the respondent used the source frequently (3 or more times per year) or not (less than 3 times per year or not at all). These seven variables are used to assess short-term trends in information consumption, but the test is imperfect: a response category was expanded in 2002. In 2000, respondents were provided with a single top category, “3 or more times,” whereas in 2002, this category was split into “3 to 5 times” and “6 or more times.” As we show below, we find pervasive increases between the two waves in the fraction seeking health information. But as a check against the possibility of an artificial increase we also explored trends in a single question whose response categories did not change between waves. In both waves, respondents were asked, “In the last 12 months, did you use the internet to look for information about a health concern or medical problem? Yes or no.” This variable, too, showed an increase between 2000 and 2002, giving us more confidence regarding the trends we observed with the other items. The primary purpose of these items, however, is not regarding trends. It is to allow us to evaluate some potential explanations for any changes in the relationship between self-rated health and mortality.

### Control Variables

All the models include the basic control variables of sex, marital status, and race (black, white, or other). In addition, some models include years of education, which serves as a potential explanatory variable given trends in average educational attainment and the role of schooling in increasing the validity of self-rated health. We do not control for income given the strong possibility of confounding with poor health, which is not shared to the same degree by education [38]. In the same vein, some models include verbal cognitive ability as a potential mechanism, measured using a 10-item test that has been included regularly in the GSS [39]. The ten items included in the GSS were selected from the twenty-one items contained in the Gallup-Thorndike verbal intelligence test [40]. In the GSS version of the test, respondents are presented with a word and asked to identify which word (or brief phrase) among five the index word comes closest in meaning to. Correct answers are then summed to create a score ranging from zero to ten. The variable has been used extensively and is conventionally referred to using its variable name, *wordsum*. Although wordsum is an imperfect measure of general intelligence, it maps well onto other research on the subject if intelligence. For instance, scores on the 10 item test have improved over time, as they have for many other tests of cognitive ability [23]. For this reason, wordsum allows us to test the possibility that self-rated health is becoming a more valid predictor of mortality because the public is more cognitively sophisticated.

### Statistical Models

The data are set in survival time fashion. To model relationships between our independent variables and the hazard of mortality we use a Cox proportional hazard model [41]. The coefficients are presented as hazard ratios, permitting a straightforward interpretation as change in the relative hazard of mortality for a unit change in the independent variables. Age does not appear as a covariate, but age is implicitly controlled in the models. It enters the estimation procedure in two ways. First, we have information about the respondents' current age, which we include as the age in which they enter the risk pool. Second, we have information about the age in which they died or were right-censored (that is, in the latter case, their age up until the last year in which the NDI linkage occurred). In this way, age information is used to construct the underlying hazard. Including age as a covariate in this context serves a different function from testing the effects of age on mortality: it provides a test of survivorship bias. Models that included age revealed some evidence for such a bias—age is associated with a *decrease* in the hazard of mortality because older survey respondents will have survived longer than younger respondents (the hazard ratio for age was .99)—but the inclusion of age did not change our central results, largely because the average age of the population is not changing much over time and the age coefficient is small. Note, however, that the survival curves produced in some models will be higher than expected, as many in the sample have already survived to age 70 or higher and some therein will also report good health, which we condition on in some of our predictions.

Our key research question pertains to a specific change over time: change in the relationship between self-rated health and mortality. For this reason, we are less interested in the average effect of self-rated health across all the years than we are in the dynamics of this effect. These changes are modeled using multiplicative interactions, in the first instance between self-rated health and year and, in later specifications, between self-rated health and the explanatory variables, including years of education, wordsum scores, and exposure to specific sources of health-related information. Year is coded as current year minus 1980, allowing the main effect of self-rated health to be interpreted as the effect of self-rated health in the first year of the series (self-rated health was not asked in 1978, making 1980 the first year). Specified in this fashion, the interaction coefficient can be interpreted as the yearly change in the relationship between self-rated health and mortality. In the final section, we model interactions between self-rated health and whether the respondent sought health information and, if so, what source he/she used. In each of these cases, we estimate two self-rated health coefficients: the first for the effects of self-rated health among those who did not use the specific source of information and the second for the effects of self-rated health among those who did. We test for differences between these coefficients using a Wald test. This alternative specification of a multiplicative interaction permits a plain interpretation with respect to the key question: whether self-rated health is a more valid predictor of mortality in one group or the other. For additional clarity, survivor functions are plotted for some key results. Table 1 presents summary statistics, as well as correlations between variables and both year and self-rated health.

**Table 1:** Descriptive Statistics, 1980–2002 General Social Survey-National Death Index Linkages with 2008 Mortality Follow-up (N = 23,307).

		Correlations with	
		Self-Rated Health	
Variable	Mean/Proportion	(1 = Excellent, 4 = Poor)	Year
Female	.57	.06	.00
White	.83	−.07	−.05
Black	.13	.07	.10
Other Race	.04	.02	−.10
Married	.56	−.06	.04
Year of Schooling	12.48	−.31	−.03
Current Smoker	.34	.06	−.07
Former Smoker	.22	.02	.04
Current Drinker	.71	−.18	.07

## Results

**Figure 2:**
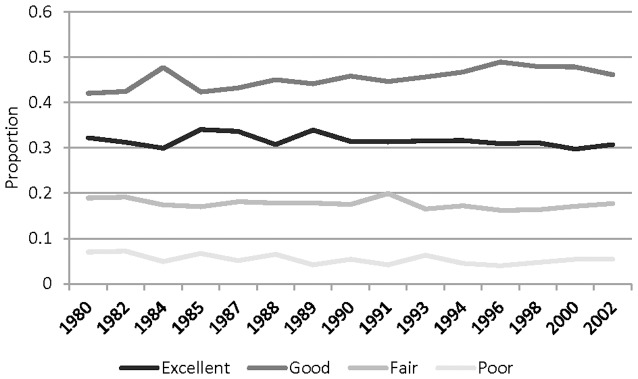
Trends in Self-Rated Health, 1980 to 2002 General Social Survey.

Figure 2 presents basic trends in self-rated health. The figure depicts the proportion reporting each of the four response categories—excellent, good, fair, and poor—over the entire period. In general, there has been some movement toward the middle. The modal response is good health, and the fraction reporting good health has increased over time. The fraction reporting excellent health, meanwhile, has declined somewhat, as has the fraction reporting poor health. This is consistent with the idea that health has objectively improved, pushing respondents out of the poor category, while expectations have improved as well, pushing respondents out of the excellent category.

**Figure 3:**
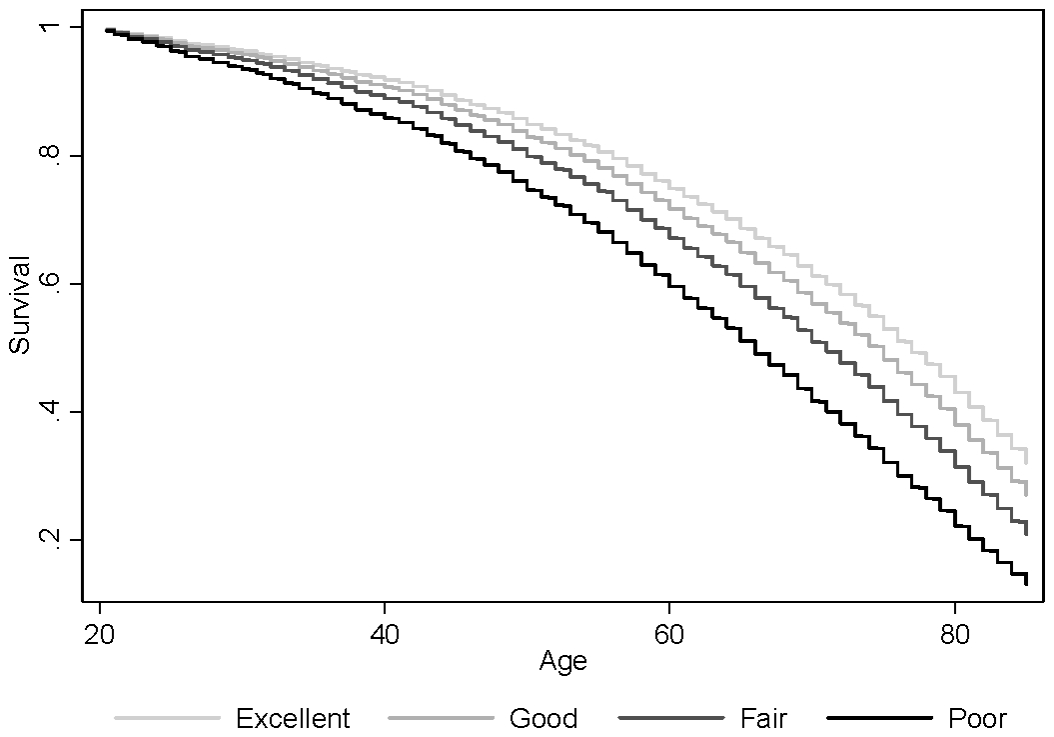
Self-Rated Health and Mortality Survival Curve, 1980–2008 General Social Survey.

With this as background, Table 2 presents an average from which the remaining results can be assessed. Model 1 explores self-rated health as a series of three dummy-variables, exhausting all the proffered categories. Model 2 estimates the three variables as sex-specific coefficients, thereby testing for sex differences. And Model 3 controls for health behaviors, exploring the sensitivity of the relationship. Model 1 reveals a strong dose-like relationship between self-rated health and mortality. The percent increase approximately doubles over each consecutive category (relative to excellent health): increasing mortality 15% for good health, 37% for fair health, and 78% for poor health. The table presents conventional significance tests—that is, of whether the ratio is significantly different from one—but supplementary Wald tests of differences between adjacent ratios reveal that each consecutive ratio is significantly larger than the one preceding it. In their magnitude, these hazard ratios are consistent with previous research. Other studies find slightly under a two-fold increase in mortality for those who report poor health relative to excellent health (see [3] for a meta-analysis). The ratios estimated here are also consistent with studies employing especially long follow-ups (of 30 years or more) [42], providing further reassurance about the GSS given its inconsistent length of follow-up between waves (a point we return to shortly). Figure 3 presents a survival curve based on the model.

**Table 2:** Cox Regression of Self-Rated Health Predicting Mortality, General Social Survey-National Death Index Linkage with 2008 Mortality Follow-up.

	Model 1	Model 2	Model 3
*Self-Rated Health (vs. Excellent)*			
Good	1.149***		1.102
	(.037)		(.059)
Fair	1.374***		1.390***
	(.056)		(.079)
Poor	1.784***		1.810***
	(.093)		(.154)
*Among Men*			
Good		1.150**	
		(.057)	
Fair		1.378***	
		(.084)	
Poor		1.593***	
		(.133)	
*Among Women*			
Good		1.149**	
		(.051)	
Fair		1.375***	
		(.071)	
Poor		1.926***	
		(.128)	
*Health Behaviors*			
Current Smoker			1.193***
			(.057)
Former Smoker			.998
			(.049)
Currently Drinks Alcohol			.978
			(.048)
Years (with 2008 mortality follow-up)	1980–2002	1980–2002	1980–1994
N	23,307	23,307	7,030
F	49.26	35.71	18.52
Prob>F	.000	.000	.000
Deaths	6,220	6,220	2,527

* p<.05;

** p<.01;

*** p<.001 (two tailed test).

*Note*: All models include controls for sex, race, education, year, and marital status. Coefficients presented as hazard ratios.

Model 2 explores sex-differences in the relationship between self-rated health and mortality. The ratios are significant for both groups, although there are no statistically significant differences between men and women. The hazard ratio for poor health is somewhat larger among women, but the difference is only marginally significant (p = .07 for a two-tailed Wald test), which is consistent with recent research [42]. Model 3 controls for risk factors. The relationship between self-rated health and mortality remains largely unchanged, although there is a slight reduction in the difference between those who report excellent health and those who report good health. Among those in fair or poor health, the relationship between self-rated health and mortality cannot be explained by poor health behaviors.

Having established an average relationship similar to those produced in other studies, Table 3 turns to the key question of our study: is the relationship between self-rated health and mortality changing over time? The table presents models that estimate this change using a linear interaction between year and each of the three self-rated health categories. This strategy allows for the possibility of discontinuous changes between levels, as might occur if most of the relevant over-time change pertains to respondents shifting between excellent and good health rather than other categories. In order to assess the sensitivity of the interactions and to rule out sources of spurious change, the models are estimated in different subsamples: initially for the full sample, then stratifying by sex, and then after deleting certain causes of death that might be implicated in trends in the validity of self-rated health. Model 1 reveals that the relationship between self-rated health and mortality is increasing, especially among those reporting fair or poor health. This increase is found among both women and men (Models 2 and 3) and is insensitive to deleting cardiovascular diseases, cancer, and external causes. Given the direction of the interaction (a growing association), the last influence is especially important insofar as accidental deaths declined between 1970 and 2002 [43] and, by nature, accidental deaths are unlikely to be anticipated by individuals and reported in self-rated health. A substantial number of accidental deaths might dilute the predictive validity of self-rated health and, thus, could explain the trend we observe. However, eliminating external causes does not change the interaction with year.

**Table 3:** Cox Regression of Self-Rated Health with Interactions Predicting Mortality, 1980–2002 General Social Survey-National Death Index Linkage with 2008 Mortality Follow-up.

	Model 1	Model 2	Model 3	Model 4	Model 5	Model 6
*Self-Rated Health (vs. Excellent)*						
Good	1.118*	1.014	1.237**	1.087	1.164*	1.098
	(.062)	(.078)	(.100)	(.072)	(.083)	(.062)
Fair	1.202**	1.095	1.360**	1.184*	1.258*	1.195*
	(.084)	(.101)	(.141)	(.095)	(.116)	(.087)
Poor	1.164	1.180	1.126	1.184	1.180	1.177
	(.100)	(.134)	(.152)	(.119)	(.134)	(.105)
*Interactions*						
Good×Year - 1980	1.004	1.014*	.993	1.008	1.001	1.005
	(.005)	(.007)	(.007)	(.006)	(.006)	(.005)
Fair×Year - 1980	1.016*	1.025**	1.005	1.022**	1.011	1.018**
	(.006)	(.008)	(.010)	(.008)	(.008)	(.007)
Poor×Year - 1980	1.050***	1.055***	1.046***	1.059***	1.052***	1.050***
	(.008)	(.010)	(.011)	(.009)	(.010)	(.008)
Year - 1980	.986***	.987*	.985*	.980***	.983**	.985***
	(.004)	(.006)	(.006)	(.005)	(.006)	(.004)

Sample	Full	Women	Men	Deleting Cancer Deaths	Deleting Cardio-vascular Deaths	Deleting External Causes
N	23,307	13,167	10,140	21,702	21,018	22,832
F	41.73	22.96	16.58	34.55	21.87	29.17
Prob>F	.000	.000	.000	.000	.000	.000
Deaths	6,220	3,362	2,858	4,615	3,931	5,745

* p<.05;

** p<.01;

*** p<.001 (two tailed test).

*Note*: All models include controls for sex, race, marital status, and education. Coefficients presented as hazard ratios.

**Figure 4:**
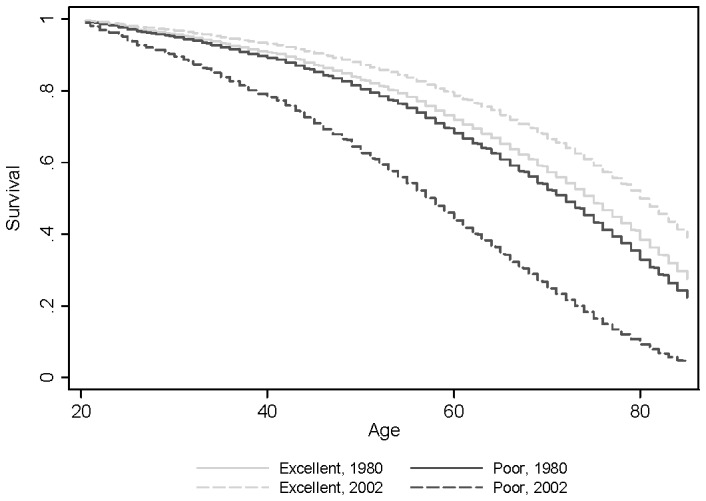
Interaction between Year and Self-Rated Health in Mortality Survival Curve, 1980–2008 General Social Survey.

For the full range of years included in the GSS—just over two decades of data—the increase in the relationship between self-rated health and mortality is not small. Figure 4 presents four survival curves, each at the edge of the observed data, for a two-by-two illustration: those in poor and excellent health in 1980 and those in poor and excellent health in 2002. The intervening years are not presented. The difference between poor and excellent in 2002 easily encompasses the difference in 1980. Expressed in terms of year-specific hazard ratios, the ratios for the 1980 baseline over progressively worse levels of self-rated health (compared to excellent health) are 1.118, 1.202, and 1.164, and for 2002, 1.221 ( = 1.118×1.004^22^), 1.704 ( = 1.202×1.016^22^), and 3.405 ( = 1.164×1.050^22^). Although the final hazard ratio (for poor health in 2002) is unusually large, it is not entirely an artifact of imposing a linear trend over a more complex series: when Model 1 is estimated in the 2002 sample alone, the hazard ratio is still 2.358.

Table 4 explores two potential mechanisms for the increase. It is possible the growing relationship between self-rated health and mortality reflects the growing cognitive sophistication of the public. In this vein, Table 4 explores whether increasing educational attainment or cognitive ability can explain the increase. Although both variables strengthen the association between self-rated health and mortality, they do not explain the increase in the relationship between self-rated health and mortality over time. Model 1 in Table 4 presents the same specification as Model 1 in Table 3, but for simplicity specifies self-rated health using a linear term rather than a series of dummy variables and adds an interaction between self-rated health and education (measured in years of completed schooling). The interaction with education is positive, indicating a growing association between self-rated health and mortality with more schooling. Furthermore, the magnitude of the interaction between self-rated health and year and the interaction between self-rated health and schooling is very similar, suggesting a parallel between the annual gain in the predictive validity of self-rated health and the unit gain associated with an additional year of schooling. The same applies to wordsum, which increases the relationship between self-rated health and mortality. In short, more intellectually sophisticated respondents provide more valid self-reports of health. We are not the first to explore whether education moderates the relationship between self-rated health and mortality, but our results add to the small set of studies finding that education increases the predictive validity of self-rated health [19], [20], [34].

**Table 4:** Cox Regression of Self-Rated Health with Mechanisms Predicting Mortality, 1980–2002 General Social Survey-National Death Index Linkage with 2008 Mortality Follow-up.

	Model 1	Model 2
Self-Rated Health	.952	.889
	(.044)	(.060)
Year – 1980	.965***	.960***
	(.006)	(.009)
Self-Rated Health×Year - 1980	1.014***	1.017***
	(.003)	(.004)
Schooling	.975*	
	(.009)	
Self-Rated Health×Schooling	1.010**	
	(.004)	
Wordsum		.920***
		(.022)
Self-Rated Health×Wordsum		1.029**
		(.010)
N	23,307	11,606
F	50.48	27.10
Prob>F	.000	.000
Deaths	6,220	3,142

* p<.05;

** p<.01;

*** p<.001 (two tailed test).

*Note*: All models include controls for sex, race, and marital status. Coefficients presented as hazard ratios.

The GSS is excellent for assessing trends in the relationship between self-rated health and mortality, but it provides little information for assessing mechanisms, especially over long stretches of time. Over shorter periods of time, however, the GSS is better. Table 5 presents variables collected in the final two waves of the GSS-NDI match. Although representing a narrow window of time, these variables allow us to assess the influence of mechanisms presumably relevant over the entire series. Table 5 presents two things. The first is the mean for each of eight health information variables, starting with whether the respondent sought health information regularly, followed by seven specific sources of health information. The fraction seeking health information increased between 2000 and 2002, rising to nearly one-quarter in 2002. The fraction using each of the seven specific sources also increased, although the largest increases were for friends/relatives and the internet.

**Table 5:** Means and Cox Regression of Self-Rated Health Predicting Mortality including Interactions with Health Information Use and Source, 2000 and 2002 General Social Survey-National Death Index Linkage with 2008 Mortality Follow-up (N = 1,729).

		*Source for Regular Information*						
	Any Source	Daily Newspaper	Magazine	Health Magazine	Doctor	Friends or Relatives	Radio or Television	Internet
*Means*								
2000	.182	.041	.051	.061	.128	.063	.046	.085
	(.011)	(.007)	(.009)	(.008)	(.011)	(.009)	(.008)	(.009)
2002	.241+	.061	.064	.089+	.170+	.103+	.068	.202+
	(.014)	(.009)	(.009)	(.009)	(.014)	(.011)	(.007)	(.013)
*Cox Models*								
Self-Rated Health×	1.377***	1.490***	1.490***	1.480***	1.419***	1.537***	1.461***	1.554***
Did Not See Information from Source	(.135)	(.130)	(.131)	(.131)	(.130)	(.132)	(.130)	(.139)
Self-Rated Health×	2.164* b	2.278*	1.776	2.155**	1.948*** a	1.438	2.418*	1.223
Did Seek Information from Source	(.358)	(.720)	(.545)	(.584)	(.388)	(.395)	(.863)	(.371)
F	5.78	5.62	5.65	5.76	5.93	5.62	6.04	6.30
Prob>F	.000	.000	.000	.000	.000	.000	.000	.000
Deaths	203	203	203	203	203	203	203	203

* p<.05;

** p<.01;

*** p<.001 (two-tailed test).

+2002 mean significantly different from 2000 mean at p<.05.

a Self-rated health hazard ratio significantly different from other self-rated health hazard ratio at p<.10.

b Self-rated health hazard ratio significantly different from other self-rated health hazard ratio at p<.05.

*Note*: A response category was expanded in the 2002 survey over the 2000 survey. See data section for a discussion. All Cox models include controls for sex, race, education, marital status, and whether or not the respondent sought any health information. Coefficients presented as hazard ratios.

**Figure 5:**
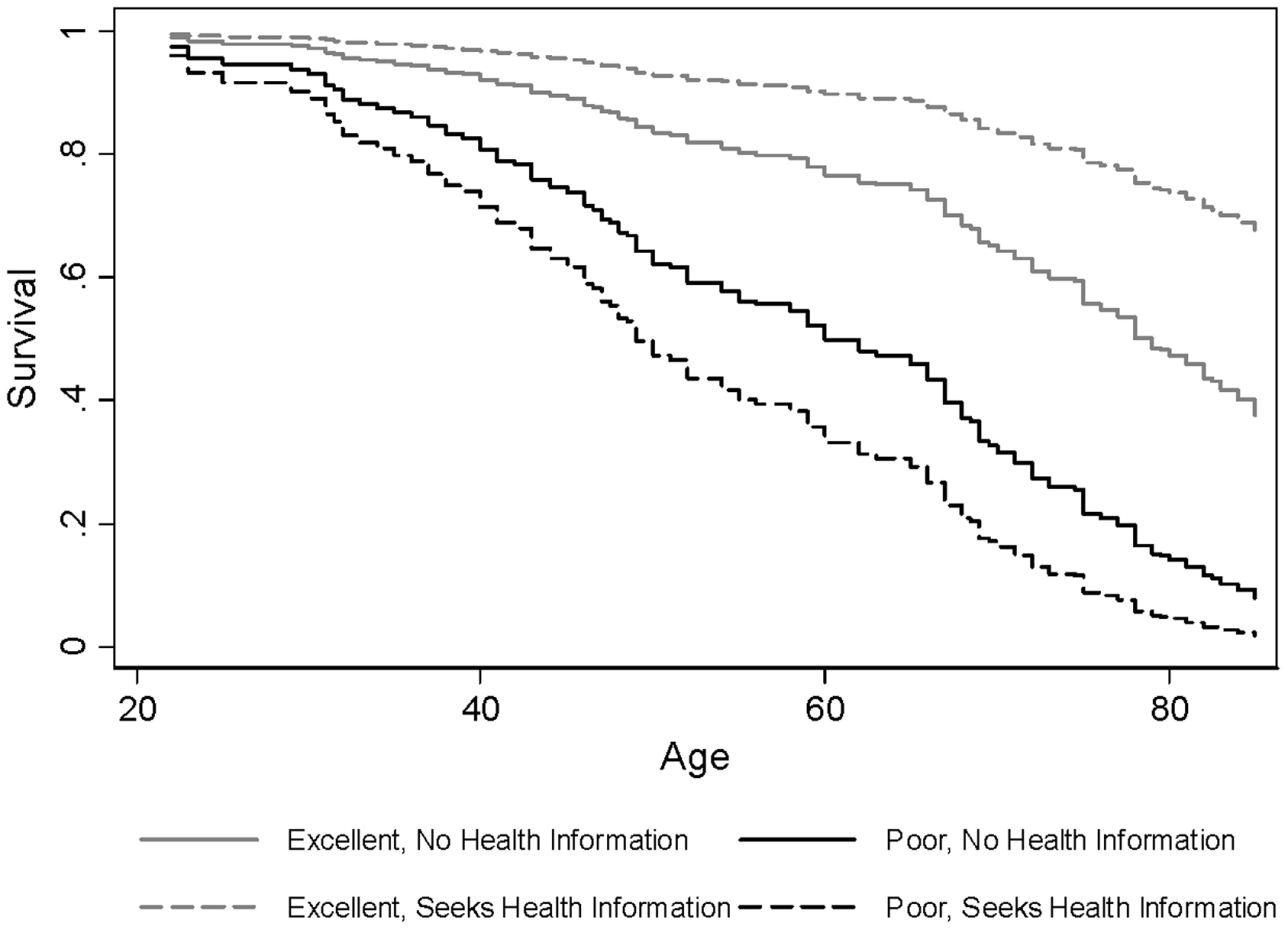
Interaction between Seeking Health Information and Self-Rated Health Represented in Mortality Survival Curve, 1980–2002 General Social Survey.

The second half of the table presents hazard ratios for self-rated health, specified as a pair of interactions, one for each value of the information-related variables. Specified in this fashion, the first hazard ratio can be interpreted as the effect of self-rated health on mortality for those who did not seek information (or consult the specific source) and the second as the effect of self-rated health among those who did. A considerably smaller sample size limits our confidence relative to the full GSS-NDI, but the patterns among the coefficients are informative for the trends observed earlier. The first column reveals that the increase in the fraction seeking health information has likely increased the relationship between self-rated health and mortality over time. The hazard ratio for self-rated health is nearly double among those who sought health-related information relative to those who did not. Figure 5 presents the survival curve for another two-by-two configuration: those in excellent and poor health and those who do and do not seek health information regularly.

The remaining columns disaggregate this pattern and explore specific sources of information. In general, consulting any specific source results in an increase in the association between self-rated health and mortality, as expected from the initial results. However, some of the increases are much larger than others and, in one case, the association is actually smaller among information-seekers. Among those who do not consult a physician, for example, each increase in self-rated health increases the hazard of mortality by 42%, whereas among those who do consult a physician, the increase is 95% (difference significant at p<.10). Among those who consult the internet, the hazard ratio is actually smaller than among those who do not consult the internet. Indeed, among those who consult the internet regularly, the association between self-rated health and mortality is statistically insignificant, whereas among those who do not consult the internet, the association remains significant, as it does on average for the entire sample. If more Americans are seeking health information exclusively on the internet, we should expect some tapering of the relationship between self-rated health and mortality. The evidence suggests, however, that Americans seek information from a variety of sources and, therefore, are simply more informed than they were in the past.

## Discussion

Self-rated health is a strong predictor of mortality and, therefore, a valid indicator of overall health. Despite speculation to the contrary, the current study shows the validity of self-rated health is increasing. Self-rated health is a much stronger predictor of mortality in 2002 than it was in 1980, leading to a wider gap in survival between those who report excellent health and those who report poor health, holding constant a variety of other influences. This increase is not small and suggests an increasingly sophisticated public, one that is learning to think more accurately about health. On this point, an illustrative comparison is with schooling. The annual increase in the predictive validity of self-rated health is equivalent to that of an additional year of education, suggesting a parallel between education and period effects. Although anticipated by some facets of the literature, this increase is not entirely expected or obvious. Indeed, if anything, research on medicalization, as well as concern about an overdiagnosed public, suggests a weakening of the relationship between self-rated health and mortality insofar as the category of poor health is admitting more superficial conditions and new conditions are constructed irrespective of their true severity with respect to mortality.

There are at least two explanations for the increase. First, it is possible that individuals are including more objective information in their self-assessments of health, allowing for a tighter coupling of subjective measures with clinically-relevant information. Second, it is possible that individuals are considering more mortality-related conditions than they did in the past. In this case, self-rated health is not necessarily more objective than it was before, but, rather, reflects a set of conditions for which a growing fraction consists of conditions related to mortality. Our results cannot separate these two explanations—one focused on better information and the other on more information—but it is useful to speculate in light of what we observe. In the abstract, the bar for good health has increased: satisfaction with health has declined over time and individuals require even better functioning than they did in the past in order to report “excellent” health [7]. On its own, this trend might increase the fraction of respondents who report fair or poor health, insofar as individuals now consider conditions that they did not consider in the past and some of these conditions are only weakly related to mortality. During the same period of time, however, the amount of mortality-relevant information has almost certainly increased too. In an exploration of cancer, for example, Viswanath [44] explores newspaper and television coverage between 1965 and 2005, finding more stories about cancer. The overall trend, then, seems to be toward more information about many conditions, rather than growing awareness of new conditions individuals were not aware of before.

Our analyses of information-seeking shed additional light on the results and further support our claim of a more informed public. For most of the sources of health-related information we measured, we find that those who seek information give more valid reports of self-rated health, meaning more exposure to health information improves predictive validity. Yet there are some notable differences between the sources that are worth exploring further, especially with respect to the internet. These differences between sources might be due either to the type of person who uses that source or to the type of information that source provides. It is possible, for example, that those who see a physician receive more objective information than those who do not, but it is also possible that more informed persons are more likely to see a physician in the first place. Similarly, some information provided on the internet might be inaccurate, but it is also possible that those who regularly consult the internet locate less reliable web-based sources. To be sure, the quality of existing online health information is generally good, but coverage is incomplete and good reading comprehension skills are required to extract the best information [45]. Another possibility is that those who seek information from different sources vary in the severity of their underlying problems. Individuals with nascent health problems, for example, might seek preliminary information on the internet, before confirming their suspicions with more reliable sources [46]. In this way, self-rated health might have a stronger relationship with mortality among those who visited a doctor than among those who used the internet, but only because the two groups represent different underlying stages of disease.

The negative influence of the internet also suggests the positive trends we observe in the predictive validity of self-rated health might not continue indefinitely. For purposes of understanding how things might change in the future, it will be important to consider the portfolio of sources individuals use and whether one source might entirely supplant another. To the extent the internet is used instead of a physician, for example, the average positive effect of seeking more information might be offset by using a less informative source. Most individuals do not rely on a single source of information, however, and different sources of information are not necessarily offsetting. Exploring public opinion trends, for example, Hesse and colleagues [47] find increasing trust both in internet-based health information and in information provided by a physician.

### Limitations

Despite its high quality, the GSS is limited in certain respects, and these limitations have implications for some of our specific claims. The GSS is a repeated cross-sectional study with mortality follow-up through 2008. The survival models used here allow for censoring, but respondents from earlier waves are naturally less likely to be censored than respondents from later waves. Moreover, studies with unusually long follow-up periods find a stronger relationship between self-rated health and mortality over the first seven to ten years of follow-up than over later years [42]. This deterioration could explain an increasing association with time insofar as the last waves of the GSS are within an increasingly narrow follow-up window. However, when we limited the sample to those with at least 10 years of follow-up (those from 1998 waves and earlier), we still find an increasing association (results not shown). We also find similar results if we limit the analysis to those who were either censored or died within 10 years. Another potential confounding influence relates to whether self-rated health reveals true health *per se* or simply reflects general life satisfaction. Bopp and colleagues [42] argue that individuals who report positive emotions and excellent health have skills to enhance their health and remain resilient, meaning the relationship between self-rated health and mortality has less to do with making an accurate assessment of health than with having health-promoting personal qualities that are merely coincident with the predictive validity of self-rated health. It is difficult to eliminate this possibility altogether, but health satisfaction, which was asked from 1980 to 1994 in the GSS, has declined over time controlling for actual self-rated health, meaning the salutogenic potential of excellent health—whatever it might be—is declining. Our focus on accuracy rather than a more general resiliency therefore seems appropriate.

Because the GSS is not a health survey, it was impossible for us to control for other chronic conditions and health behaviors. A common procedure in the literature on self-rated health and mortality is to demonstrate that self-rated health predicts mortality above and beyond controls for a wide variety of more specific and objective indicators. Although we were unable to do the same in the GSS, it is not clear whether doing so would change our main conclusion, as our goal was not to demonstrate the predictive validity of self-rated health, but rather to assess whether that validity has changed over time. Controlling for other risk factors would certainly reduce the association between self-rated health and mortality in any given year, but it would not clearly do the same for the interaction between self-rated health and year. Indeed, it could increase the slope of the growing association in a suppressor fashion if “poor” health includes more chronic conditions that are only weakly associated with mortality. Alternatively, if introducing these controls did reduce the interaction, one interpretation is entirely consistent with the one we already provide: that the public is demonstrating a stronger association between their subjective reports of health and more objective indicators because they are more informed. Further work in the vein of mechanisms would be useful, but research on trends in the relationship between self-rated health and mortality need not proceed in exactly the same way as research on the cross-sectional relationship between self-rated health and mortality.

## Conclusion

Researchers have long recognized the value of self-rated health as an indicator of overall health. Self-rated health is fundamentally subjective, but it stands as a uniquely strong predictor of mortality and, thus, provides a pedestal upon which the psychosocial approach to health rests. This study adds to the literature in finding that the predictive value of self-rated health is increasing. It also, we hope, illustrates the value of thinking about how individuals evaluate their own health and, by extension, how individuals are subject to the cultural currents surrounding them. The predictive validity of self-rated health is strong and growing. For this reason, there is even less reason to discount the subjective assessments of individuals when it comes to understanding their health. Yet there is much to be learned at the still imperfect intersection of self-rated health and mortality.
